# Global Burden of HIV among Men Who Engage in Transactional Sex: A Systematic Review and Meta-Analysis

**DOI:** 10.1371/journal.pone.0103549

**Published:** 2014-07-28

**Authors:** Catherine E. Oldenburg, Amaya G. Perez-Brumer, Sari L. Reisner, Jason Mattie, Till Bärnighausen, Kenneth H. Mayer, Matthew J. Mimiaga

**Affiliations:** 1 Department of Epidemiology, Harvard School of Public Health, Boston, Massachusetts, United States of America; 2 The Fenway Institute, Fenway Community Health, Boston, Massachusetts, United States of America; 3 Department of Sociomedical Sciences, Columbia Mailman School of Public Health, New York, New York, United States of America; 4 Department of Global Health and Population, Harvard School of Public Health, Boston, Massachusetts, United States of America; 5 Africa Centre for Health and Population Science, Mtubatuba, South Africa; 6 Department of Medicine, Beth Israel Deaconess, Boston, Massachusetts, United States of America; 7 Department of Psychiatry, Massachusetts General Hospital, Boston, Massachusetts, United States of America; The University of New South Wales, Australia

## Abstract

**Background:**

Men who engage in transactional sex, the exchange of sex for money, goods, or other items of value, are thought to be at increased risk of HIV, but there have been no systematic attempts to characterize HIV burden in this population. We undertook a systematic review and meta-analysis to quantify the burden in this population compared with that of men in the general population to better inform future HIV prevention efforts.

**Methods:**

We searched seven electronic databases, national surveillance reports, and conference abstracts for studies of men who engage in transactional sex published between 2004–2013. Random effects meta-analysis was used to determine pooled HIV prevalence and prevalence ratios (PR) for the difference in HIV prevalence among men who engage in transactional sex as compared to general population men.

**Findings:**

Of 66 studies included representing 31,924 men who had engaged in transactional sex in 28 countries, pooled biological assay-confirmed HIV prevalence was 10.5% (95% CI = 9.4 to 11.5%). The highest pooled HIV prevalence was in Sub-Saharan Africa (31.5%, 95% CI = 21.6 to 41.5%), followed by Latin America (19.3%, 95% CI = 15.5 to 23.1%), North America (16.6%, 95% CI = 3.7 to 29.5%), and Europe (12.2%, 95% CI = 6.0 to 17.2%). Men who engaged in transactional sex had an elevated burden of HIV compared to the general male population (PR = 20.7, 95% CI = 16.8 to 25.5).

**Conclusions:**

The global burden of HIV is disproportionately high among men who engage in transactional sex compared with the general male population. There is an urgent need to include this population in systematic surveillance as well as to scale-up access to quality HIV prevention programs.

## Introduction

Transactional sex, or the exchange of sex for money, goods, drugs, or other items of value (including protection, housing, or food), is thought to be associated with an increased vulnerability to HIV. Men who engage in transactional sex are considered a subpopulation of the men who have sex with men (MSM) community, but current research indicates that they may have differential HIV risk compared to MSM who do not engage in transactional sex, although there is conflicting evidence as to whether engagement in transactional sex is associated with increased risk-taking behavior. Some studies have shown that MSM who engage in transactional sex may have a higher prevalence of HIV than those who do not report transactional sex. [1]–[3] Drivers of both engagement in transactional sex and HIV risk among this population are complex and vary by geographic location. For example, motivations for engaging in transactional sex may be different in regions where homosexuality and/or sex work is criminalized. Structural factors, such as low educational attainment and few opportunities for gainful employment, may motivate transactional sex. [4] However, evidence from regions such as Australia, that have relatively less MSM stigma, suggests that transactional sex may also be a form of expression of sexuality. [4] There are also likely large regional differences in risk-taking behavior and transactional sex that are reflective of local culture and environments.

Psychosocial factors that may increase HIV risk in this population include an increased burden of depression and mental health distress [5], [6], sexual violence [6], a history of childhood sexual abuse [6], and substance use disorders. [1], [6] In particular, substance use may encourage men to engage in transactional sex and may be a motivator for continuing to rely on income from transactional sex. Men who engage in transactional sex may have differential condom use patterns with commercial and non-commercial partners, which can increase risk for HIV. [3], [7]–[10] Finally, clients of men who engage in transactional sex may offer greater monetary incentive for unprotected sex, or may react violently if men insist on using a condom. [11].

Men who engage in transactional sex span the sexual orientation spectrum, and a large proportion may identify as bisexual or heterosexual, with wide-ranging estimates from different settings of 30 to 75%. [8], [12] Some men who engage in transactional sex do so with women in addition to, or instead of, other men. Those who engage in transactional sex primarily with women may have different risk as compared to men who engage in transactional sex primarily with men. HIV prevention programming that aims to recruit homosexual/gay-identified men only may not reach non-gay identified or “closeted” men who sell sex. In addition, men who engage in transactional sex primarily with men may be married to women or have female primary and/or casual sexual partners [3], [13]–[18], and may act as an HIV transmission “bridge” to the general population.

To characterize the worldwide burden of HIV among men who engage in transactional sex, we undertook a systematic review of the literature and meta-analysis to assess the distribution of HIV prevalence across the globe and to assess the prevalence of HIV as compared to the general male population. An increased understanding of the burden of HIV among men who engage in transactional sex, including quantifying the burden of HIV in this population globally compared with that of other men, is crucial to understanding the evolving nature of the HIV epidemic and informing corresponding policy and HIV prevention efforts.

## Methods

### Search Strategy

This meta-analysis was conducted and reported according to Preferred Reporting Items for Systematic Reviews and Meta-Analyses (PRISMA) Guidelines (Checklist S1). We searched seven electronic databases for studies published between January 1, 2004 and July 31, 2013 (to identify articles published in the preceding 10-year period) including PubMed, EMBASE, PsycINFO, Sociological Abstracts, POPLine, CINAHL, and Web of Science. Search terms related to transactional sex included “commercial sex”, “sex work*”, “male sex worker*”, “prostitution”, “exchange sex”, and “transactional sex”. In addition, we performed a search including the terms “HIV” and “men who have sex with men”. We also searched abstracts from the International AIDS Society (IAS), the American Public Health Association (APHA), the Conference on Retroviruses and Opportunistic Infections (CROI), and the International Society for Sexually Transmitted Disease Research (ISSTDR). HIV surveillance reports including demographic and health surveys (DHS) and integrated biological and behavioral surveillance (IBBS) reports were also searched. Reference lists of all included articles were reviewed for additional articles.

### Inclusion and Exclusion Criteria

Studies were included if they contained primary, quantitative data on HIV prevalence among men (individuals assigned a male sex at birth and presently identified as a male/man) who reported exchanging any sex act for anything of value, including money, goods, or drugs. Studies were included regardless of whether HIV status was determined by laboratory methods or via self-report. Studies published in English, Spanish, French, or Portuguese, or if enough study information was published in an English-language abstract, were included. For studies that reported results from overlapping cohorts, the study with the most complete data was included in the review and meta-analysis. Studies reporting the larger sample size, or in a peer-reviewed journal versus an abstract or surveillance report were considered the “more complete” study. In cases in which no delineation was made between reporting HIV prevalence among male and transgender male-to-female sex workers, the study was included if the majority (≥80%) of participants in the study were not transgender male-to-female. Three studies were excluded because they did not differentiate men from transgender women in HIV estimates [19]–[21].

### Data Extraction

Data were extracted independently on a standardized data collection form by two separate reviewers (CEO, APB, and/or JM) with >90% agreement. Adjudication for inconsistencies was done through discussion and if necessary a third reviewer (SLR) served as the tiebreaker. Extracted data included total number of men who reported transactional sex in the study, total number of men who were tested for HIV or self-reported their HIV serostatus, and total number testing or reporting HIV infection. Additional data extracted included sampling methodology, study design, definition of transactional sex (categorized as male sex worker, exchanged sex for money at least once in the previous 12 months, and ever exchanged sex for money), country, and region, and if the study reported individual-level factors that increase HIV risk including drug use (injection or non-injection), engagement in unprotected anal sex (UAS) with commercial and/or noncommercial partners, clinical depression, experience of childhood sexual abuse (CSA), and sexually transmitted infection (STI) prevalence.

### Data Analysis

Meta-analysis using a DerSimonian-Laird random effects model [22] was used to generate an overall pooled point estimate and 95% confidence interval for HIV prevalence separately by biological assay and by self-report for all eligible studies, and then restricted to studies which reported ≥50 participants. After the initial assessment of HIV prevalence by self-report and of any sample size, all analyses included only studies that assessed HIV prevalence by biological assay and reported ≥50 participants. Pooled point estimates of biological assay-confirmed HIV prevalence were calculated by country, region, and definition of transactional sex (“male sex workers”, “exchanged sex in the last 12 months”, and “ever exchanged sex”). A random effects model was used to allow for heterogeneity between studies. [22] Random effects meta-regression was used to assess differences in HIV prevalence by definition of transactional sex. Publication bias was assessed with Egger’s test [23] and Begg’s test. [24] Methodological quality of the studies was determined using a modified GRADE scoring assessing sampling methodology, study design, HIV measure, if the study population was well-defined, and generalizability of the study (Table S1). [25], [26].

Prevalence ratios comparing the prevalence of biological assay-confirmed HIV among men reporting engagement in transactional sex to the prevalence of HIV among men aged 15 and over in the general population were calculated to assess the excess burden of HIV associated with transactional sex among men. HIV prevalence estimates for men of reproductive age were calculated using UNAIDS 2009 data for the total estimated number of HIV cases as the numerator and the United States Census Bureau International Division data for the total number of men aged 15 and over in the global population in 2009 as the denominator. [27] The midpoint of the uncertainty bounds for number of HIV-infected males aged 15 and over was used as the estimate of absolute number of HIV-infected males in China because no estimates for the absolute number were available in the UNAIDS 2009 report. We chose the 2009 report since the studies represented in this analysis were published between 2004–2013, and thus this provided a midpoint. Prevalence ratios were calculated with a random-effects model. A standard correction of 0.5 was added to any zero cells. Heterogeneity was assessed with an I^2^ and τ^2^ statistics, by region of study origin and overall. All analyses were conducted in Stata 12.0 (StataCorp, College Station, TX).

## Results

**Figure 1:**
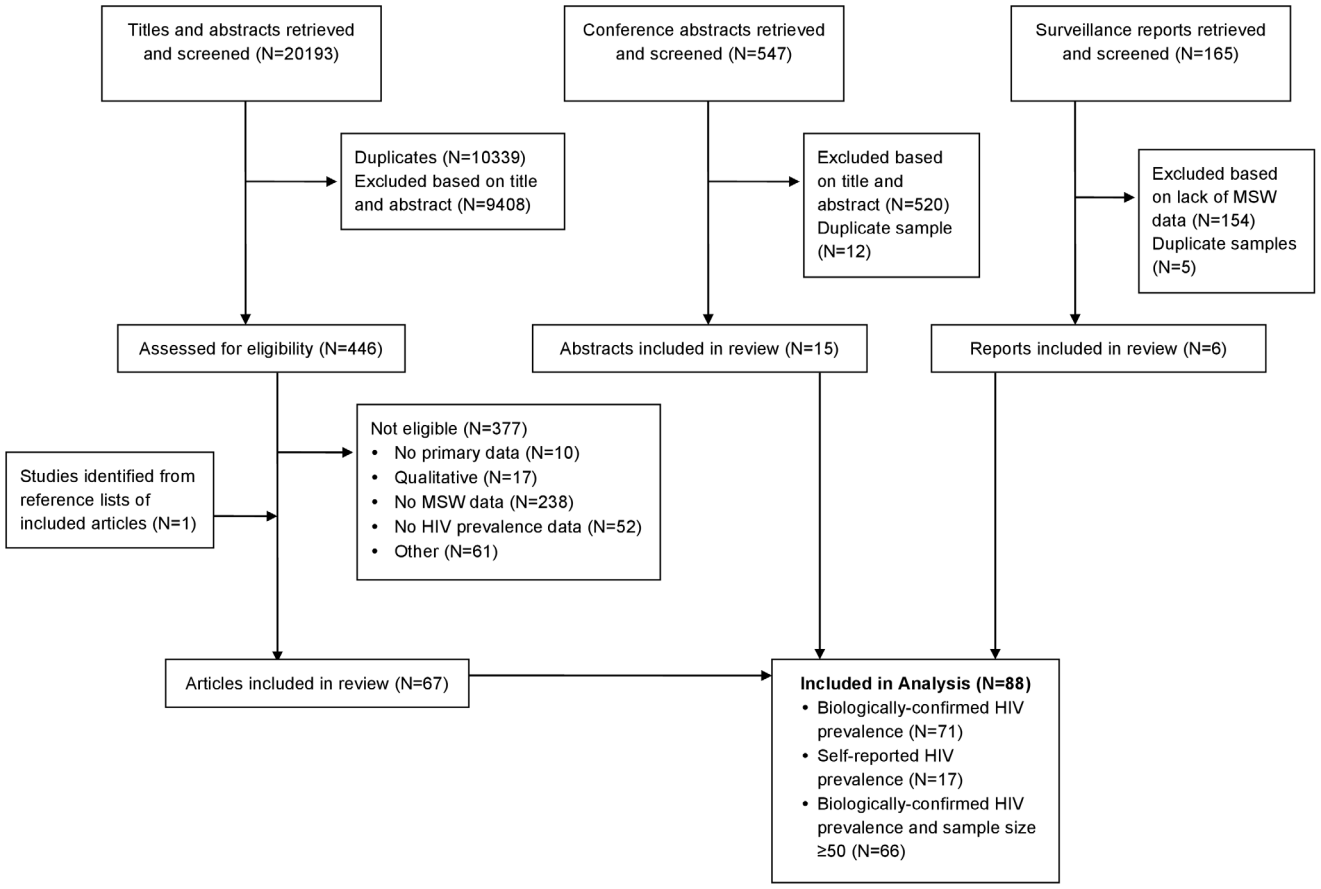
Flow diagram of included studies.

Of 20,193 titles and abstracts, 547 conference abstracts, and 165 surveillance reports, 446 titles and abstracts were selected for further review, and a total of 88 articles, abstracts, or surveillance reports representing 34,531 individuals in 30 countries were included in the review. Of the 88 studies, 14 were in East Asia (all of which were from China)[12], [13], [28]–[39], 16 in South Asia [3], [16], [17], [40]–[52], 15 in Southeast Asia [7], [9], [15], [53]–[64], 11 in Latin America [2], [14], [18], [65]–[72], 9 in Sub-Saharan Africa [73]–[81], 9 in Europe [8], [10], [82]–[88], and 14 in North America [1], [5], [89]–[99]. Of these, 66 [1]–[3], [7]–[9], [12], [14]–[17], [28], [29], [31]–[39], [41]–[51], [53], [55], [57], [59]–[61], [63]–[67], [70]–[72], [74]–[77], [79]–[88], [93]–[95], [100] included a biological HIV assay and had a sample size of ≥50, and thus were included in the analyses, representing 31,924 men who engaged in transactional sex with other men in 28 countries (Figure 1).

Table 1 shows the pooled HIV prevalence among men who engaged in transactional sex by HIV measurement and sample size. The HIV prevalence among men engaging in transactional sex across all 88 studies was 11.9% (95% CI = 10.9 to 12.9%). Across 71 studies reporting biological assay-confirmed HIV serostatus, including small samples, HIV prevalence was 10.7% (95% CI 9.7 to 11.8%). Across 66 studies with biological assay and restricted to studies with ≥50 participants, the pooled HIV prevalence was 10.5% (95% CI 9.4% to 11.5%).

**Table 1:** Pooled HIV prevalence by HIV measurement and sample size.

	N (k)	Pooled HIV Prevalence (95% CI)
Overall	34,531 (88)	11.9% (10.9 to 12.9%)
Biological assay	32,007 (71)	10.7% (9.7 to 11.8%)
Self-report, untested or unknown status excluded from calculation	2,524 (17)	20.6% (14.5 to 26.8%)
Self-report, untested or unknown status assumed to be negative	3,109 (17)	13.3% (9.6 to 17.0%)
Biological assay, ≥50 participants in study	31,924 (66)	10.5% (9.4 to 11.5%)

N = number of subjects; k = number of studies; 95% CI = 95% Confidence Interval.

**Figure 2:**
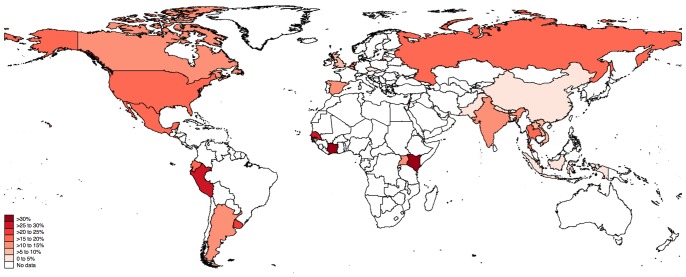
Pooled HIV prevalence by country among studies reporting biologically-confirmed HIV prevalence with a sample size of ≥50 (N = 66).

The pooled biological assay-confirmed HIV prevalence among men who engaged in transactional sex by region, country, and definition of transactional sex is presented in Table 2. By region, the highest pooled HIV prevalence was in Sub-Saharan Africa (31.5%, 95% CI 21.6 to 41.5%), followed by Latin America (19.3%, 95% CI 15.5 to 23.1%), North America (16.6%, 95% CI 3.7 to 29.5%), and Southeast Asia (12.9%, 95% CI 8.8 to 17.0%). The lowest prevalence region was South Asia (2.7%, 95% CI 1.7 to 3.6%). Figure 2 graphically depicts HIV prevalence by country. Overall heterogeneity of the pooled estimates was high (τ^2^ = 2800.08). Begg’s test (*P* = 0.002) and Egger’s test (*P*<0.001) indicated the possibility of publication bias, which could be due to small studies or high heterogeneity. With the exception of South Asia, these tests suggested no publication bias in analyses stratified by geographic region.

**Table 2:** Pooled HIV prevalence among men who engage in transactional sex by region, country, and definition of transactional sex.

	Overall (95% CI)^1^		Male sex workers		Exchanged sex in last 12 months		Ever exchanged sex		*P* 2
	N (k)	HIV Prevalence (95%CI)	N (k)	HIV Prevalence (95%CI)	N (k)	HIV Prevalence (95%CI)	N (k)	HIV Prevalence (95%CI)	
**East Asia** (China)	**7,221 (12)**	**4.1% (2.8 to 5.4%)**	**2,200 (7)**	**4.7% (2.5 to 6.8%)**	**5,001 (5)**	**3.7% (1.7 to 5.7%)**	**–**	**–**	**0.64**
**South Asia**	**14,453 (14)**	**2.7% (1.7 to 3.6%)**	**11,433 (11)**	**1.0% (0.5 to 1.5%)**	**809 (1)**	**2.0% (1.0 to 2.9%)**	**2,211 (2)**	**11.8% (6.0 to 17.6%)**	**0.0001**
Bangladesh	985 (1)	0.0% (0.0 to 0.0%)	985 (1)	0.0% (0.0 to 0.0%)	–	–	–	–	–
India	2,211 (2)	11.8% (6.0 to 17.6%)	100 (2)	24.0% (15.6 to 32.4%)	–	–	2,211 (2)	11.8% (6.0 to 17.6%)	–
Nepal	218 (2)	3.5% (1.0 to 5.9%)	218 (2)	3.5% (1.0 to 5.9%)	–	–	–	–	–
Pakistan	11,039 (9)	1.2% (0.6 to 1.7%)	10,230 (8)	1.0% (0.5 to 1.6%)	809 (1)	2.0% (1.0 to 2.9%)	–	–	–
**Southeast Asia**	**4,477 (12)**	**12.9% (8.8 to 17.0%)**	**3,292 (8)**	**12.6% (8.1 to 17.1%)**	**778 (2)**	**13.2% (4.3 to 22.0%)**	**407 (2)**	**13.9% (0.0 to 41.1%)**	**0.98**
Indonesia	250 (1)	3.6% (1.3 to 5.9%)	250 (1)	3.6% (1.3 to 5.9%)	–		–	–	–
Laos	119 (1)	8.4% (3.4 to 13.4%)	–	–	119 (1)	8.4% (3.4 to 13.4%)	–	–	–
Thailand	3,201 (7)	17.5% (13.7 to 21.2%)	2,211 (5)	15.6% (11.6 to 19.6%)	659 (1)	17.5% (14.6 to 20.3%)	484 (2)	23.2% (13.9 to 32.5%)	–
Vietnam	907 (3)	6.8% (0.0 to 13.8%)	831 (2)	9.9% (2.8 to 17.0%)	–	–	76 (1)	0.0% (0.0 to 0.0%)	–
**Latin America**	**1,704 (9)**	**19.3% (15.5 to 23.1%)**	**1,416 (6)**	**20.4% (15.5 to 25.2%)**	**–**	**–**	**288 (3)**	**16.7% (18.7 to 15.1%)**	**0.46**
Argentina	170 (2)	11.2% (6.4 to 15.9%)	114 (1)	11.4% (5.6 to 17.2%)	–	–	56 (1)	10.7% (2.6 to 18.8%)	–
Ecuador	76 (1)	19.7% (10.8 to 28.7%)	–	–	–	–	76 (1)	19.7% (10.8 to 28.7%)	–
El Salvador	156 (1)	19.2% (13.0 to 25.4%)	–	–	–	–	156 (1)	19.2% (13.0 to 25.4%)	–
Mexico	507 (2)	17.2% (12.0 to 22.5%)	507 (2)	17.2% (12.0 to 22.5%)	–	–	–	–	
Peru	478 (2)	27.3% (20.2 to 34.4%)	478 (2)	27.3% (20.2 to 34.4%)	–	–	–	–	–
Uruguay	317 (1)	21.8% (17.2 to 26.3%)	317 (1)	21.8% (17.2 to 26.3%)	–	–	–	–	–
**Sub-Saharan Africa**	**1,608 (7)**	**31.5% (21.6 to 41.5%)**	**1,386 (5)**	**36.3% (24.9 to 47.6%)**	**93 (1)**	**26.9% (17.9 to 35.9%)**	**129 (1)**	**12.4% (6.7 to 18.1%)**	**0.34**
Cote d’Ivoire	96 (1)	50.0% (40.0 to 60.0%)	96 (1)	50.0% (40.0 to 60.0%)	–	–	–	–	–
Kenya	1,290 (4)	33.2% (21.0 to 45.3%)	1,290 (4)	33.2% (21.0 to 45.3%)	–	–	–	–	–
Senegal	93 (1)	26.9 (17.9 to 35.9%)	–	–	93 (1)	26.9 (17.9 to 35.9%)	–	–	–
Uganda	129 (1)	12.4% (6.7 to 18.1%)	–	–	–	–	129 (1)	12.4% (6.7 to 18.1%)	–
**Europe**	**1,854 (8)**	**10.2% (5.2 to 15.2%)**	**988 (6)**	**12.2% (9.1 to 15.3%)**	**636 (1)**	**9.3% (7.0 to 11.5%)**	**230 (1)**	**0.9% (0.0 to 2.1%)**	**0.05**
Belgium	120 (1)	10.8% (5.3 to 16.4%)	120 (1)	10.8% (5.3 to 16.4%)	–	–	–	–	–
Czech Republic	230 (1)	0.9% (0.0 to 2.1%)	–	–	–	–	230 (1)	0.9% (0.0 to 2.1%)	–
Israel	53 (1)	5.7% (0.0 to 11.9%)	53 (1)	5.7% (0.0 to 11.9%)	–	–	–	–	–
Netherlands	99 (1)	11.1% (4.9 to 17.3%)	99 (1)	11.1% (4.9 to 17.3%)	–	–	–	–	–
Russia	50 (1)	18.0% (7.4 to 28.6%)	50 (1)	18.0% (7.4 to 28.6%)	–	–	–	–	–
Spain	666 (2)	14.2% (9.6 to 18.8%)	666	14.2% (9.6 to 18.8%)	–	–	–	–	–
United Kingdom	636 (1)	9.3% (7.0 to 11.5%)	–	–	636 (1)	9.3% (7.0 to 11.5%)	–	–	–
**North America**	**607 (4)**	**16.6% (3.7 to 29.5%)**	–	–	**108 (1)**	**26.9% (18.5 to 35.2%)**	**499 (3)**	**13.4% (0.0 to 27.3%)**	**0.43**
United States	349 (2)	19.3% (8.0 to 30.6%)	–	–	–	–	349 (2)	19.5% (13.7 to 25.3%)	–
Canada	258 (2)	14.1% (0.0 to 38.4%)	–	–	108 (1)	26.9% (18.5 to 35.2%)	150 (1)	2.0% (0 to 4.2%)	–
**OVERALL**	**31,924 (66)**	**10.5% (9.4 to 11.5%)**	**20,715 (43)**	**10.4% (9.2 to 11.5%)**	**7,445 (11)**	**8.4% (5.7 to 11.1%)**	**3,917 (12)**	**12.6% (7.4 to 17.8%)**	**0.78**

1 Analyses restricted to individual studies reporting ≥50 participants and biological assay-confirmed HIV prevalence;

2 Type-3 *P*-value from random effects meta-regression adjusted for country-level HIV prevalence among males aged 15+ (presented for regions).

N = number of subjects; k = number of studies; 95% CI = 95% Confidence Interval.

Table 3 presents prevalence ratios for biological assay-confirmed HIV prevalence among men engaging in transactional sex compared to the general population of males age 15 or older. The pooled prevalence ratio (PR) for HIV infection for men who engage in transactional sex compared to the general population of men aged 15 and older was 20.7 (95% CI 16.8 to 25.5). The regions with the highest PRs included East Asia (PR 51.3, 95% CI 38.5 to 68.2), Latin America (PR 35.0, 95% CI 27.5 to 44.7), and Europe (PR 32.4, 95% CI 25.9 to 40.6). The overall heterogeneity of the prevalence ratio estimates was high (I^2^ = 96.9%, τ^2^ = 2076.67). Begg’s test (*P* = 0.18) and Egger’s test (*P* = 0.89) suggested no evidence of publication bias.

**Table 3:** Prevalence ratios comparing pooled HIV prevalence among men who engage in transactional sex to general population men aged 15 and older.

	N (k)	HIV Prevalence^1^(95%CI)	Country-Wide HIVPrevalence, Males 15+	I^2^	Prevalence Ratio^2^(95% CI)
**East Asia** (China)	**7,221 (12)**	**4.1% (2.8 to 5.4%)**	0.10%	**73.4%**	**51.3 (38.5 to 68.2)**
**South Asia**	**14,453 (14)**	**2.7% (1.7 to 3.6%)**		**95.0%**	**12.3 (7.1 to 21.2)**
Bangladesh	985 (1)	0.0% (0.0 to 0.0%)	0.0093%		5.5 (0.3 to 87.3)
India	2,211 (3)	11.8% (6.0 to 17.6%)	0.34%		34.4 (20.6 to 57.6)
Nepal	218 (2)	3.5% (1.0 to 5.9%)	0.46%		8.2 (4.2 to 16.2)
Pakistan	11,039 (9)	1.2% (0.6 to 1.7%)	0.11%		11.0 (6.1 to 19.8)
**Southeast Asia**	**4,477 (12)**	**12.9% (8.8 to 17.0%)**		**83.9%**	**15.3 (12.6 to 18.5)**
Indonesia	250 (1)	3.6% (1.3 to 5.9%)	0.24%		14.7 (7.7 to 27.9)
Laos	119 (1)	8.4% (3.4 to 13.4%)	0.25%		33.5 (18.5 to 60.7)
Thailand	3,201 (7)	17.5% (13.7 to 21.2%)	1.21%		14.4 (11.8 to 17.7)
Vietnam	907 (3)	6.8% (0.0 to 13.8%)	0.59%		12.7 (5.3 to 30.5)
**Latin America**	**1,704 (9)**	**19.3% (15.5 to 23.1%)**		**83.1%**	**35.0 (27.5 to 44.7)**
Argentina	170 (2)	11.2% (6.4 to 15.9%)	0.53%		21.2 (13.9 to 32.4)
Ecuador	76 (1)	19.7% (10.8 to 28.7%)	0.51%		39.0 (24.8 to 61.4)
El Salvador	156 (1)	19.2% (13.0 to 25.4%)	1.10%		17.5 (12.7 to 24.1)
Mexico	507 (2)	17.2% (12.0 to 22.5%)	0.42%		41.0 (30.2 to 55.8)
Peru	478 (2)	27.3% (20.2 to 34.4%)	0.56%		49.2 (37.9 to 63.9)
Uruguay	317 (1)	21.8% (17.2 to 26.3%)	0.55%		39.7 (32.2 to 49.0)
**Sub-Saharan Africa**	**1,608 (7)**	**31.5% (21.6 to 41.5%)**		**97.7%**	**8.9 (5.4 to 14.5)**
Cote d’Ivoire	96 (1)	50.0% (40.0 to 60.0%)	2.56%		19.5 (16.0 to 23.8)
Kenya	1,290 (4)	33.2% (21.0 to 45.3%)	4.60%		6.9 (4.7 to 10.0)
Senegal	93 (1)	26.9 (17.9 to 35.9%)	0.69%		38.7 (27.7 to 54.1)
Uganda	129 (1)	14.2% (7.7 to 20.6%)	5.81%		2.4 (1.5 to 3.8)
**Europe**	**1,854 (8)**	**12.2% (6.0 to 17.2%)**		**52.5%**	**32.4 (25.9 to 40.6)**
Belgium	120 (1)	10.8% (5.3 to 16.4%)	0.23%		46.4 (27.8 to 77.6)
Czech Republic	230 (1)	0.9% (0.0 to 2.1%)	0.033%		26.5 (6.7 to 105.4)
Israel	53 (1)	5.7% (0.0 to 11.9%)	0.20%		27.7 (9.2 to 83.2)
Netherlands	99 (1)	11.1% (4.9 to 17.3%)	0.24%		46.3 (26.5 to 80.8)
Russia	50 (1)	18.0% (7.4 to 28.6%)	0.89%		20.3 (11.2 to 36.6)
Spain	666 (2)	14.2% (9.6 to 18.8%)	0.52%		27.6 (20.0 to 38.2)
United Kingdom	636 (1)	9.3% (7.0 to 11.5%)	0.23%		39.6 (31.1 to 50.5)
**North America**	**607 (4)**	**16.6% (3.7 to 29.5%)**		**93.9%**	**24.6 (11.3 to 53.7)**
United States	349 (2)	19.3% (8.0 to 30.6%)	0.77%		24.6 (13.3 to 45.3)
Canada	258 (2)	14.1% (0.0 to 38.4%)	0.38%		19.8 (0.67 to 581.5)
**OVERALL**	**31,924 (66)**	**10.5% (9.4 to 11.5%)**		**96.9%**	**20.7 (16.8 to 25.5)**

1 Among studies confirming HIV infection with a biological assay and among studies reporting ≥50 participants;

2 All prevalence ratios significant (*P*<0.05) except for Bangladesh and Canada.

N = number of subjects; k = number of studies; I^2^ = variation in pooled prevalence ratio due to heterogeneity; 95% CI = 95% Confidence Interval.

The distribution of studies reporting individual risk factors related to HIV vulnerability by region is shown in Table 4. Reporting of individual risk factors varied widely by type of risk factor. The majority of studies reported UAS (77.3% among men who engage in transactional sex); less than half of studies reported substance use, injection drug use, and STI history or current infection (43.2%, 43.2%, and 48.9% respectively). Few studies reported depression (8.0%) or childhood sexual abuse (12.5%). The only risk factor whose reporting differed significantly by region among men who engaged in transactional sex was injection drug use (*P*<0.001), with most studies in North America including injection drug use history as a risk behavior (78.6%), and few in East Asia (7.1%) and Latin America (9.1%).

**Table 4:** Proportion of studies reporting individual factors that affect vulnerability to HIV by region, overall and specifically among men who engage in transactional sex.

	Total Number of Studies	Any Drug Use	Injection Drug Use	Depression	Childhood Sexual Abuse	Unprotected Anal Sex	STI^1^ History/Prevalence
**East Asia (China)**	14						
Overall^2^		8 (57.1%)	4 (28.6%)	2 (14.3%)	2 (14.3%)	14 (100%)	12 (85.7%)
Transactional Sex^3^		4 (28.6%)	1 (7.1%)	2 (14.3%)	2 (14.3%)	12 (85.7%)	7 (50.0%)
**South Asia**	16						
Overall^2^		6 (37.5%)	11 (68.8%)	0 (0%)	1 (6.3%)	16 (100%)	12 (75.0%)
Transactional Sex^3^		6 (37.5%)	10 (62.5%)	0 (0%)	1 (6.3%)	15 (93.8%)	10 (62.5%)
**Southeast Asia**	15						
Overall^2^		10 (66.7%)	6 (40.0%)	1 (6.7%)	0 (0%)	14 (93.3%)	10 (66.7%)
Transactional Sex^3^		8 (53.3%)	6 (40.0%)	1 (6.7%)	0 (0%)	13 (86.7%)	7 (46.7%)
**Latin America**	11						
Overall^2^		7 (63.6%)	2 (18.2%)	1 (9.1%)	1 (9.1%)	9 (81.8%)	8 (72.7%)
Transactional Sex^3^		2 (18.2%)	1 (9.1%)	1 (9.1%)	1 (9.1%)	7 (63.6%)	4 (36.4%)
**Sub-Saharan Africa**	9						
Overall^2^		6 (66.7%)	5 (55.6%)	1 (11.1%)	1 (11.1%)	9 (100%)	6 (66.7%)
Transactional Sex^3^		4 (44.4%)	3 (33.3%)	0 (0%)	1 (11.1%)	7 (77.8%)	4 (44.4%)
**Europe**	9						
Overall^2^		5 (55.6%)	6 (66.7%)	0 (0%)	2 (22.2%)	5 (55.6%)	6 (66.7%)
Transactional Sex^3^		5 (55.6%)	6 (66.7%)	0 (0%)	2 (22.2%)	5 (55.6%)	6 (66.7%)
**North America**	14						
Overall^2^		10 (71.4%)	12 (85.7%)	3 (21.4%)	4 (28.6%)	10 (71.4%)	6 (42.9%)
Transactional Sex^3^		9 (64.3%)	11 (78.6%)	3 (21.4%)	4 (28.6%)	9 (64.3%)	5 (35.7%)
**Total**	88						
Overall^2^		52 (59.1%)	46 (52.3%)	8 (9.1%)	11 (12.5%)	77 (87.5%)	60 (68.2%)
Transactional Sex^3^		38 (43.2%)	38 (43.2%)	7 (8.0%)	11 (12.5%)	68 (77.3%)	43 (48.9%)
**P** 4 **-value**	–						
Overall^2^		0.61	0.005	0.45	0.29	0.003	0.39
Transactional Sex^3^		0.25	<0.001	0.33	0.29	0.18	0.69

1 Including syphilis, gonorrhea, chlamydia, human papilloma virus (HPV), and/or herpes simplex virus (HSV);

2 Risk factor reported among any population in the study (i.e., men who have sex with men);

3 Risk factor reported specifically among men who engage in transactional sex;

4 Fisher’s exact test comparing frequency of reporting factors by geographic region of study, overall and among men who report transactional sex.

A single study reported HIV incidence, among men who engage in transactional sex in Thailand, and found an incidence of 7.4 per 100 person-years (95% CI 4.7–11.1). [64].

## Discussion

The burden of HIV infection is disproportionately high among men who engage in transactional sex globally, with these men having more than 20 times the prevalence of HIV infection as compared to the general male population. These data underscore the urgent global need to scale up access to quality HIV prevention programs specific to this population. Previous systematic reviews have demonstrated a disproportionate burden of HIV among female sex workers (FSW), transgender women, and MSM as compared to the general population. [101]–[104] The findings of the current study indicate that men who engage in transactional sex bear a similarly higher burden of HIV. Drivers of engagement in transactional sex are multifaceted, and include drug use, economic motivation, stigma, harassment and discrimination from multiple sources and levels of influence, likely impact the health and well-being (e.g., can lead to increased depression and lower self-acceptance) of men who engage in transactional sex and may limit access to and uptake of HIV prevention and treatment services. [105] Moreover, resulting distress may reduce their ability to uptake and incorporate HIV prevention counseling information and behavioral skills, leading to continued sexual risk. Ensuring that this vulnerable population is included in strategic plans and resource allocation to address both structural and individual levels of influence is essential to adequately curb the spread of HIV globally.

These results demonstrate a consistently disproportionate burden of HIV globally among men who engage in transactional sex as compared to men in the general population. Regions in which the HIV epidemic is largely driven by sexual transmission and concentrated among MSM had the largest disparities in the burden of HIV. In Latin America, North America, and Europe, regions in which the HIV epidemic is concentrated among MSM [106], both absolute and relative measures of HIV burden among men engaging in transactional sex were high. The absolute HIV prevalence in Latin America was nearly 20%, and was second only to Sub-Saharan Africa. The regions with the third and fourth-highest HIV prevalence were North America and Europe, respectively. Despite these regions being culturally, socially, economically, and politically diverse, these data show a consistently higher burden of HIV among men who engage in transactional sex. While North America and Europe were the only regions that represented higher-income countries in this study, their inequitable burden was second only to that of China and Latin America, underscoring the association of the social marginalization of sex work with conditions that potentiated HIV spread. Importantly, men who engage in transactional sex should not be overlooked in national HIV prevention strategies in countries of a wide range of economic development.

Sub-Saharan Africa had the highest absolute HIV prevalence among men who engage in transactional sex, with an estimated pooled HIV prevalence of nearly one-third, but this region also had the lowest pooled prevalence ratio of all regions included in this meta-analysis. This is reflective of the generalized background HIV epidemic of many countries in that region. Male-male sexual behavior is highly stigmatized and often criminalized in many Sub-Saharan African countries, and in countries such as Uganda there are extreme penalties for engaging in male-male sex. [107], [108] These penalties limit access to healthcare and HIV prevention services among MSM, including men who engage in transactional sex with other men, thereby increasing vulnerability to HIV, and late entry into care leading to increased spread because of uncontrolled viremia (i.e. higher community viral load). [107], [108] These men may also engage in transactional sex with women, and may represent a substantially different population than those who engage in transactional sex with men.

China was the only country in East Asia represented in this study, and had the largest disparity in HIV prevalence among men who engage in transactional sex as compared to the general population, with more than 50 times the risk of HIV infection. However, China had a lower pooled HIV prevalence than any region except South Asia. China has recently experienced evidence of a growing HIV epidemic among MSM, and previous studies have demonstrated elevated HIV prevalence among men who sell sex to men as compared to MSM who do not sell sex. [109] This result demonstrates a need to enhance the HIV prevention response among this population in China.

In an effort to report relevant data on those who do and do not identify as a sex worker, we used a broad definition of transactional sex in this meta-analysis, and included both male sex workers (MSWs) who self-identify as sex workers and engage in transactional sex regularly, as well as men who engage in transactional sex occasionally and may not identify as such. Men who engage in transactional sex more often or with more partners may have a greater HIV risk as compared to those who exchange sex less frequently. [2] Studies reported HIV prevalence by a variety of definitions and temporal periods in which participants had engaged in transactional sex. An inherent difficulty in assessing differences using these definitions is the degree to which there is overlap among them. Individual studies may have lumped “male sex workers” in the “ever exchange sex” category, which could not be distinguished in this analysis and may have made the results appear not to differ by strata. Reliance on self-report may affect estimates, since sex work is highly stigmatized, so some individuals in these studies may have engaged in it, but were unwilling to disclose, or others might only have admitted a lesser level of activity. Finally, the source of HIV infection in this population is not necessarily due to commercial partnerships, which could mitigate the importance of definition of transactional sex. This review was unable to analyze source of HIV infection. However, in total, the results of this review point to the need for standardized definitions of sex work and transactional sex, including identification of reliable measures of engaging in transactional sex and/or buying sex.

Consideration of individual-level risk factors that affect biological and behavioral vulnerability to HIV is critically needed to understand how HIV prevention interventions may best serve the needs of this population. Studies reporting individual risk factors such as unprotected anal sex (UAS) reported very different definitions of UAS (i.e., with the last partner, any in the last 3 months, with commercial versus non-commercial partners), and as such could not be meta-analyzed. While most studies reported UAS among men who engaged in transactional sex, fewer than half of studies reported STIs or substance use; very few studies reported psychosocial factors such as depression and childhood sexual abuse, which can have sequelae that lead to increased risk for HIV. [6], [110] Future studies should consider these syndemic (i.e., co-occurring and potentially interacting) factors within cultural contexts, and future prevention interventions should consider specifically how these syndemic conditions increase vulnerability to HIV and how prevention interventions can best mitigate risk. Interestingly, there was a statistically significant geographic difference in reporting injection drug use. Studies in North America, Europe, and South Asia in particular were significantly more likely to report injection drug use, whereas studies in Latin America much less frequently reported injection drug use. Given that engagement in transactional sex may be driven by drug use, and injection drug use is a strong risk factor for HIV, characterizing the degree of this behavior will be important to better understand the epidemic. In addition, in general studies less frequently reported risk factors for HIV infection among men who engaged in transactional sex specifically as compared to reporting among MSM overall. Some of this was likely driven by the fact that many of these studies were primarily focused on MSM, and reported transactional sex secondarily. Despite this, to fully understand the dynamics of the HIV epidemic in this population, a better characterization of factors that influence HIV transmission among men who engage in transactional sex will be necessary.

There are some limitations to consider in this analysis. The pooled HIV prevalence estimates likely are biased by inherent difficulties in accessing and sampling men who engage in transactional sex. The degree of stigma, harassment and discrimination that this population faces in many contexts, which includes physical threats and extreme punishments in some cases, may limit research and surveillance of this key risk group in many global settings. There were few studies from regions in which male-male sexual behavior is illegal with very strong punishments. Sampling strategies varied widely, and could have influenced the results. In addition, it is likely that modality of meeting commercial partners has changed over time, with an increasing reliance on the Internet and mobile phones. Some evidence suggests that men who meet commercial partners violence in the Internet have differential HIV risk as compared to those who meet partners in physical venues (i.e., street-based or other physical venues). [5] We were unable to analyze HIV prevalence trends by partner-meeting modality, since typically HIV estimates were not reported by venue. However, attention should be given HIV prevention needs for men who meet commercial partners via the Internet, as they may differ substantially than those who meet partners in physical venues.

Pooled HIV prevalence could be underestimated if lower-risk individuals are more accessible and therefore more likely to be included in research and surveillance. These results may also have been affected by publication bias, which could result in over- or underestimates of pooled HIV prevalence as well as biased prevalence ratios. This study included only estimates that have been published in the peer-reviewed scientific literature or country-level surveillance data. Ultimately, the quality and accuracy of the pooled estimates is limited by the quality and accuracy of the data from which they arose. Restricting analyses only to studies reporting biologically-confirmed HIV prevalence with sample sizes of greater than 50 likely improves the quality and generalizability of these data. We used general population HIV prevalence among men as the comparison group as opposed to HIV prevalence among MSM, due to the lack of systematic surveillance of HIV among MSM which precluded comparable comparison. Furthermore, men who engage in transactional sex may be included in general population estimates, which could bias the results of corresponding prevalence ratios. Despite these limitations, this study presents the first comprehensive global overview of the current status of literature relating to the HIV epidemic among men who engage in transactional sex with other men, providing important evidence for the urgent need to scale up HIV prevention interventions among this group.

This study demonstrates a consistent elevation in HIV burden among men who engage in transactional sex globally in comparison with men in the general population. An understanding of populations of individuals who engage in transactional sex would be improved by routinely asking about transactional sex history in surveillance studies in diverse settings, which would allow for better monitoring of and response to the epidemic. The results of this study provide a macroscopic view of the state of the literature on this population, and demonstrate a need for additional studies worldwide, and in particular demonstrates gaps in knowledge in regions where MSM behavior is more highly stigmatized. This study also underscores the need for development of structural interventions, including economic interventions that may reduce reliance on income from transactional sex, as well as individual-level interventions such as increased access to substance use treatment. While drivers of the HIV epidemic differ geographically within countries and regions, the disproportionate burden of HIV shouldered by this population clearly demonstrates a need for a multisectoral response to increase efforts in HIV surveillance, research, and prevention, as well as increased allocation of resources for such a response, in this highly vulnerable yet understudied group.

## Ethics Statement

An ethics statement was not required for this work.

## Supporting Information

Table S1 **Modified GRADE table describing quality of included studies.**
(DOCX)

Checklist S1 **PRISMA Checklist.**
(DOCX)

Protocol S1 **Study protocol.**
(PDF)
