# Supplementary material for: Global Burden of HIV among Men Who Engage in Transactional Sex: A Systematic Review and Meta-Analysis
Source: PLoS One. 2014 Jul 28;9(7):e103549. doi: 10.1371/journal.pone.0103549 (PMC4113434; doi:10.1371/journal.pone.0103549)
Supplement: Table S1 — Modified GRADE table describing quality of included studies. (DOCX) [file pone.0103549.s001.docx]

**Table S1.** Modified GRADE score assessing quality of included studies

| **Study** | **Sample Size** | **N HIV-Infected** | **Design**  +4 for prospective or cross-sectional  +2 for retrospective | **Sampling Methodology**  -1 if non-probability  -2 if not reported | **HIV measure**  -1 if self-report | **Report Individual Risk Characteristics**  -1 if no report of HIV risk factors for transactional sex | **Defined population**  -1 if no reported  inclusion/  exclusion criteria | | **Generalizable^1^**  -1 if serious issues  -2 if very serious issues | **Total Score** |
| --- | --- | --- | --- | --- | --- | --- | --- | --- | --- | --- |
| **ARTICLES** | | | | | | | | | | |
| Bacon et al | 154 | 21 | 4 | -1 | 0 | 0 | 0 | | -1 | 2 |
| Ballester et al | 91 | 1 | 4 | -1 | -1 | 0 | 0 | | 0 | 2 |
| Baral et al, 2011 | 24 | 11 | 4 | -1 | 0 | -1 | 0 | | -2 | 0 |
| Baral et al, 2010 | 50 | 9 | 4 | -1 | 0 | 0 | 0 | | 0 | 3 |
| Belza et al | 418 | 51 | 4 | -1 | 0 | 0 | -1 | | 0 | 2 |
| Bokhari et al | 809 | 16 | 4 | 0 | 0 | 0 | -1 | | 0 | 3 |
| Bimbi et al | 48 | 8 | 4 | -1 | -1 | 0 | 0 | | 0 | 2 |
| Brahmam et al | 2023 | 293 | 4 | 0 | 0 | 0 | 0 | | 0 | 4 |
| Bruckova et al | 230 | 2 | 4 | -1 | 0 | 0 | 0 | | 0 | 3 |
| Burnette et al | 557 | 46 | 4 | 0 | -1 | 0 | -1 | | -1 | 1 |
| Cai et al | 394 | 21 | 4 | 0 | 0 | 0 | 0 | | 0 | 4 |
| Chan et al | 985 | 0 | 4 | -2 | 0 | -1 | -1 | | -1 | -1 |
| Chemnasiri et al | 312 | 46 | 4 | 0 | 0 | 0 | 0 | | 0 | 4 |
| Cheng et al | 151 | 17 | 4 | -1 | 0 | 0 | -1 | | 0 | 2 |
| Clatts et al | 25 | 23 | 4 | 0 | -1 | 0 | 0 | | -1 | 2 |
| Cohan et al | 119 | 19 | 4 | -1 | -1 | 0 | -1 | | 0 | 1 |
| Colby et al | 76 | 0 | 4 | 0 | 0 | 0 | 0 | | 0 | 4 |
| Creswell et al | 156 | 30 | 4 | 0 | 0 | -1 | 0 | | 0 | 3 |
| dos Ramos Farias et al | 114 | 13 | 4 | -1 | 0 | 0 | 0 | | 0 | 3 |
| Feng et al | 118 | 13 | 4 | -1 | 0 | -1 | 0 | | 0 | 2 |
| Fujimoto | 179 | 50 | 4 | -1 | -1 | 0 | -1 | | 0 | 1 |
| Guadamuz et al | 181 | 14 | 4 | -1 | 0 | 0 | -1 | | 0 | 2 |
| Gupta et al | 13 | 7 | 4 | -1 | 0 | -1 | 0 | | -2 | 0 |
| Haley et al | 150 | 3 | 4 | -1 | 0 | 0 | 0 | | -1 | 2 |
| Hawkes et al | 646 | 1 | 4 | 0 | 0 | 0 | 0 | | 0 | 4 |
| He et al | 100 | 4 | 4 | 0 | -1 | 0 | 0 | | 0 | 3 |
| Hernandez et al | 188 | 16 | 4 | -1 | 0 | -1 | 0 | | 0 | 2 |
| Hladik et al | 129 | 16 | 4 | 0 | 0 | -1 | 0 | | 0 | 3 |
| Jacoboson et al | 76 | 15 | 4 | 0 | 0 | -1 | 0 | | 0 | 3 |
| Kral et al | 195 | 49 | 4 | -1 | 0 | 0 | 0 | | -1 | 2 |
| Kuyper et al | 108 | 29 | 4 | -1 | 0 | 0 | -1 | | -1 | 1 |
| Lama et al | 349 | 85 | 4 | -1 | 0 | -1 | 0 | | 0 | 2 |
| Leuridan et al | 120 | 13 | 2 | -1 | 0 | 0 | -1 | | 0 | 0 |
| Li et al | 659 | 115 | 4 | 0 | 0 | 0 | 0 | | 0 | 4 |
| Liu et al | 418 | 14 | 4 | 0 | 0 | 0 | -1 | | 0 | 3 |
| Mimiaga et al | 32 | 10 | 4 | -1 | -1 | 0 | 0 | | -1 | 1 |
| MMWR | 754 | 118 | 4 | 0 | 0 | 0 | 0 | | 0 | 4 |
| Montano et al | 317 | 69 | 4 | -1 | 0 | -1 | 0 | | 0 | 2 |
| Mor et al | 53 | 3 | 4 | 0 | 0 | 0 | 0 | | 0 | 4 |
| Morton | 197 | 54 | 4 | -1 | -1 | 0 | 0 | | 0 | 2 |
| Ngo et al | 190 | 0 | 4 | -1 | 0 | 0 | 0 | | -1 | 2 |
| Nguyen et al | 9 | 3 | 4 | 0 | 0 | -1 | -1 | | -2 | 0 |
| Pisani et al | 250 | 9 | 4 | -1 | 0 | 0 | -1 | | 0 | 2 |
| Pitpitan et al | 240 | 19 | 4 | -1 | -1 | 0 | -1 | | -1 | 0 |
| Prado Cortez | 41 | 7 | 4 | 0 | -1 | 0 | 0 | | -1 | 2 |
| Ruan et al | 112 | 0 | 4 | 0 | 0 | -1 | -1 | | 0 | 2 |
| Schuelter-Trevisol et al | 13 | 6 | 4 | -1 | 0 | 0 | 0 | | -2 | 1 |
| Segura et al | 56 | 6 | 4 | -1 | 0 | -1 | 0 | | 0 | 2 |
| Sethi et al | 636 | 59 | 4 | -1 | 0 | 0 | 0 | | 0 | 3 |
| Shaw et al | 1532 | 8 | 4 | 0 | 0 | 0 | 0 | | 0 | 4 |
| Sheridan et al | 119 | 10 | 4 | 0 | 0 | -1 | 0 | | 0 | 3 |
| Shinde et al | 24 | 4 | 4 | -1 | 0 | 0 | 0 | | -2 | 1 |
| Smith et al | 26 | 1 | 4 | -1 | -1 | 0 | -1 | | -1 | 0 |
| Tao et al | 118 | 6 | 4 | 0 | 0 | 0 | 0 | | 0 | 4 |
| Timpson et al | 152 | 39 | 4 | -1 | -1 | 0 | 0 | | 0 | 2 |
| Toledo et al | 414 | 78 | 4 | 0 | 0 | 0 | 0 | | 0 | 4 |
| van der Elst et al | 343 | 67 | 4 | -1 | 0 | 0 | -1 | | -1 | 1 |
| van Griensven et al | 331 | 92 | 4 | -1 | 0 | -1 | 0 | | 0 | 2 |
| Vuylsteke et al | 96 | 48 | 4 | -1 | 0 | 0 | 0 | | 0 | 3 |
| Wade et al | 93 | 25 | 4 | 0 | 0 | 0 | 0 | | 0 | 4 |
| Washington et al | 43 | 28 | 4 | -1 | -1 | 0 | 0 | | -1 | 1 |
| Williams et al | 347 | 69 | 4 | 0 | -1 | 0 | 0 | | -1 | 2 |
| Wong et al | 100 | 4 | 4 | 0 | -1 | 0 | 0 | | 0 | 3 |
| Wu e t al | 4358 | 203 | 4 | -1 | 0 | 0 | 0 | | 0 | 3 |
| Xiao et al | 295 | 10 | 4 | 0 | 0 | 0 | 0 | | 0 | 4 |
| Zhang et al | 138 | 3 | 4 | 0 | 0 | -1 | 0 | 0 | | 3 |
| Zhao et al | 850 | 67 | 4 | 0 | 0 | 0 | 0 | | 0 | 4 |
| **ABSTRACTS** | | | | | | | | | | |
| Altaf et al, 2006 | 800 | 20 | 4 | -2 | 0 | 0 | -1 | 0 | | 1 |
| Altaf et al, 2008 | 199 | 14 | 4 | 0 | 0 | 0 | -1 | 0 | | 3 |
| Colby et al, 2012 | 227 | 13 | 4 | -1 | 0 | 0 | -1 | 0 | | 2 |
| Colby et al, 2010 | 300 | 19 | 4 | -1 | -1 | 0 | -1 | 0 | | 1 |
| Cuypers et al | 99 | 11 | 2 | -1 | 0 | 0 | -1 | 0 | | 2 |
| Del Romero Guerrero et al | 248 | 42 | 4 | -1 | 0 | -1 | 0 | 0 | | 2 |
| Gakii et al | 164 | 78 | 4 | -1 | 0 | 0 | -1 | 0 | | 2 |
| Gayet et al | 284 | 42 | 4 | 0 | 0 | 0 | -1 | 0 | | 3 |
| Kladsawad et al | 550 | 114 | 4 | 0 | 0 | 0 | -1 | 0 | | 3 |
| Konda et al | 129 | 41 | 4 | -1 | 0 | 0 | -1 | 0 | | 2 |
| Magis et al | 223 | 45 | 4 | 0 | 0 | 0 | -1 | 0 | | 3 |
| McKinnon et al | 510 | 206 | 4 | -1 | 0 | 0 | -1 | 0 | | 2 |
| Muraguri et al | 273 | 71 | 4 | 0 | 0 | 0 | -1 | 0 | | 3 |
| Saleem et al | 400 | 5 | 4 | 0 | 0 | 0 | -1 | 0 | | 3 |
| Yu et al | 79 | 7 | 4 | -2 | 0 | 0 | 0 | 0 | | 2 |
| **IBBS** | | | | | | | | | | |
| Nepal 2007 | 135 | 4 | 4 | 0 | 0 | 0 | 0 | 0 | | 4 |
| Nepal 2004 | 83 | 4 | 4 | 0 | 0 | 0 | 0 | 0 | | 4 |
| Pakistan 2011 | 3674 | 60 | 4 | 0 | 0 | 0 | 0 | 0 | | 4 |
| Pakistan 2008 | 1200 | 9 | 4 | 0 | 0 | 0 | 0 | 0 | | 4 |
| Pakistan 2005 | 1779 | 8 | 4 | 0 | 0 | 0 | 0 | 0 | | 4 |
| Vietnam 2009 | 531 | 72 | 4 | 0 | 0 | 0 | 0 | 0 | | 4 |

^1^Issues related to generalizability include: small sample size of men who engage in transactional sex (<50 “serious”, <25 “very serious”), inclusion/exclusion criteria and make this population not generalizable to men who engage in transactional sex in that geographic region, lack of reporting of recruitment strategy; Note: Sample size and total number of HIV-infected are not included in the grade score
