# Supplementary material for: Global Burden of HIV among Men Who Engage in Transactional Sex: A Systematic Review and Meta-Analysis
Source: PLoS One. 2014 Jul 28;9(7):e103549. doi: 10.1371/journal.pone.0103549 (PMC4113434; doi:10.1371/journal.pone.0103549)
Supplement: Protocol S1 — Study protocol. (PDF) [file pone.0103549.s003.pdf]

STUDY PROTOCOL

Global burden of HIV among men who engage in transactional sex: a systematic review and meta-analysis

MOTIVATION

Men who have sex with men (MSM) have a greater risk of being infected with HIV compared to the general population in many settings.[1] Female sex workers experience a disproportionately high burden of HIV globally.[2] While sex work in and of itself may not be a behavioral risk factor for HIV, male sex workers (MSWs) may be at increased vulnerability to HIV through a variety of mechanisms, such as access to prevention services, condom negotiation with commercial and non-commercial partners, and stigma and discrimination.

The global burden of HIV infection among MSWs is not well understood, and there is a paucity of evidence regarding risk of HIV in this group. This protocol describes a systematic review and meta-analysis to assess the global prevalence and determinants of HIV infection among MSWs.

SEARCH STRATEGY

Search Terms

1. Commercial sex
2. Sex work*
3. Male sex workers
4. Men who have sex with men
5. HIV

Synonyms to be searched across databases:

Commercial sex work: commercial sex; transactional sex; prostitution

Sex worker: commercial sex worker; transactional sex worker; prostitute

Specifically, searches will be performed as follows:

PubMed

commercial sex[tiab] OR sex work*[tiab] OR male sex worker*[tiab] OR transactional sex[tiab] OR prostitution [tiab] OR exchange sex[tiab] AND ("2004/01/01"[PDAT] : "2013/12/31"[PDAT])

Ebsco

TI ("commercial sex" OR "sex work*" OR "male sex worker*" OR "transactional sex" OR "prostitution " OR "exchange sex") OR AB ("commercial sex" OR "sex work*" OR "male sex worker*" OR "transactional sex" OR "prostitution " OR "exchange sex")

ProQuest

TI("commercial sex" OR "sex work*" OR "male sex worker*" OR "transactional sex" OR "prostitution " OR "exchange sex") OR AB("commercial sex" OR "sex work*" OR "male sex worker*" OR "transactional sex" OR "prostitution " OR "exchange sex")

Web of Science

TS=("commercial sex" OR "sex work*" OR "male sex worker*" OR "transactional sex" OR "prostitution " OR "exchange sex")

Databases:

- MEDLINE
- EMBASE
- PsycINFO
- Web of Science
- CINAHL
- POPLine
- Sociological Abstracts

Other Sources:

- National surveillance system data reports
    - Demographic health surveys
    - Biobehavioral surveillance studies
- Abstract databases
    - CROI
    - APHA
    - IAS
    - ISSTDR

INCLUSION CRITERIA

- Primary, quantitative data
- Includes HIV prevalence data on biologically male sex workers (defined as any sex act in exchange for anything of value, such as good or money) who have male and/or female commercial partners
- All methods of measurement of HIV prevalence will be included (both biological testing and self-reported HIV positivity)
- Articles published in English, Spanish, Portuguese, or French OR enough information in an English abstract for inclusion

Dates of inclusion: All abstracts, reports, etc published from January 1, 2004 to present

For studies that report results from overlapping cohorts, the study with the most complete data will be included (larger sample sizes that report more outcomes will be considered favorably).

OUTCOMES

Primary:

- HIV prevalence

Secondary:

- Unprotected anal sex (UAS)
- Serodiscordant unprotected anal sex (SDUAS)

DATA EXTRACTION

The following variables will be extracted onto standardized data collection forms:

- Study type (cross-sectional, cohort, RCT, etc)
- Sample size
- Geographic location of study (region, country, city)
  - For multisite studies, data will be recorded overall and by study site
- Method of HIV ascertainment (self report, biological assay)
- HIV prevalence (total number of HIV+ MSWs/total MSWs in study)
- Proportion of MSWs engaging in UAS and SDUAS
- Injection drug use
- Depression
- Childhood sexual abuse

ANALYSES

Analysis of HIV prevalence will be conducted by:

- Overall prevalence for all included studies

- By region (Southeast Asia, South Asia, East Asia, Sub-Saharan Africa, Middle East and North Africa, Latin America and the Caribbean, Eastern Europe, Western Europe, North America [United States and Canada])
- By epidemic level (generalized, concentrated, low-level)
- By country
- Odds Ratios comparing HIV prevalence among MSWs compared to the general male population (reproductive age) and MSM

Estimates will be pooled using a DerSimonian-Laird random effects model for overall HIV prevalence and prevalence by the above subgroups. Odds ratios for HIV prevalence among MSWs compared to MSM will be calculated using the Mantel-Haenszel method with a random effects model. A τ^2 statistic will be reported to assess between-study heterogeneity. Analyses will be conducted in Stata 12.0.

References

1 Baral S, Sifakis F, Cleghorn F, Beyrer C. Elevated risk for HIV infection among men who have sex with men in low- and middle-income countries 2000-2006: A systematic review. *Elevated risk for HIV infection among men who have sex with men in low- and middle-income countries 2000-2006: A systematic review* 2007; **4**: e339.

2 Baral S, Beyrer C, Muessig K, *et al.* Burden of HIV among female sex workers in low-income and middle-income countries: a systematic review and meta-analysis. *The Lancet Infectious Diseases* 2012; **12**: 538–49.
